# Muscle forces acting on the greater trochanter lead to a dorsal warping of the apophyseal growth plate

**DOI:** 10.1111/joa.13944

**Published:** 2023-09-11

**Authors:** Christian Max Ziegler, Ferdinand Wagner, Karoline Alleborn, Tobias Geith, Boris Michael Holzapfel, Bernhard Heimkes

**Affiliations:** ^1^ Department of Orthopaedics and Trauma Surgery, Musculoskeletal University Center Munich (MUM), LMU University Hospital Ludwig‐Maximilians‐Universität München Munich Germany; ^2^ Department of Pediatric Surgery, Dr. von Hauner Children's Hospital Ludwig‐Maximilians‐University Munich Munich Germany; ^3^ Institute of Health and Biomedical Innovation, Queensland University of Technology (QUT) Queensland Brisbane Australia; ^4^ Department of Interventional Radiology Technical University of Munich Munich Germany; ^5^ Klinikum Dritter Orden, Department of Pediatric Surgery Pediatric Orthopedic and Neuroorthopaedic Section Munich Germany

**Keywords:** AY‐angle, crocodile sign, form follows function, trochanter major growth plate warp

## Abstract

The apophyseal growth plate of the greater trochanter, unlike most other growth plates of the human body, exhibits a curved morphology that results in a divergent pattern resembling an open crocodile mouth on plain antero‐posterior radiographs. To quantify the angular alignment of the growth plate and to draw conclusions about the function of the muscles surrounding it, we analyzed 57 MRI images of 51 children and adolescents aged 3–17 years and of six adults aged 18–52 years. We measured the angulation of the plate relative to the horizontal plane (AY angle) and the trajectories of the muscles attaching to the greater trochanter of the proximal femur. From anterior to posterior, the AY angle shows a decrease of 33.44°. In the anterior third, the cartilage is angled at a mean of 51.64°, and in the posterior third, the mean angulation is 18.6°. This indicates that the cartilage in the anterior region of the greater trochanteric apophysis is subject to more vertically oriented force vectors compared to the posterior region, as the growth plates align perpendicular to the force vectors acting on them. Combining the measured muscle trajectories with the physiological cross‐sectional areas (PCSA) available from the literature revealed that, in addition to the known internal and external lateral traction ligament systems, a third, dorsally located traction ligament system exists that may be responsible for the dorsal deformation of the AY angle.

## Graphical abstract

The apophyseal growth plate of the greater trochanter exhibits a curved morphology that results in a divergent pattern resembling an open crocodile mouth on radiographs. To quantify the angular alignment of the growth plate and to draw conclusions about the function of the muscles surrounding it, we measured the angulation of the plate relative to the horizontal plane and the trajectories of the muscles attaching to the greater trochanter of the proximal femur.

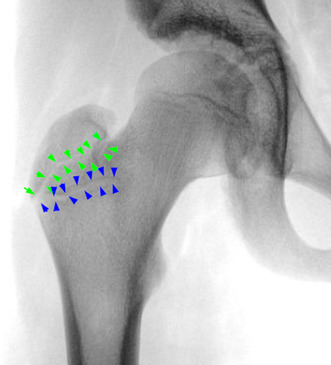

## 1 INTRODUCTION

**FIGURE 1:**
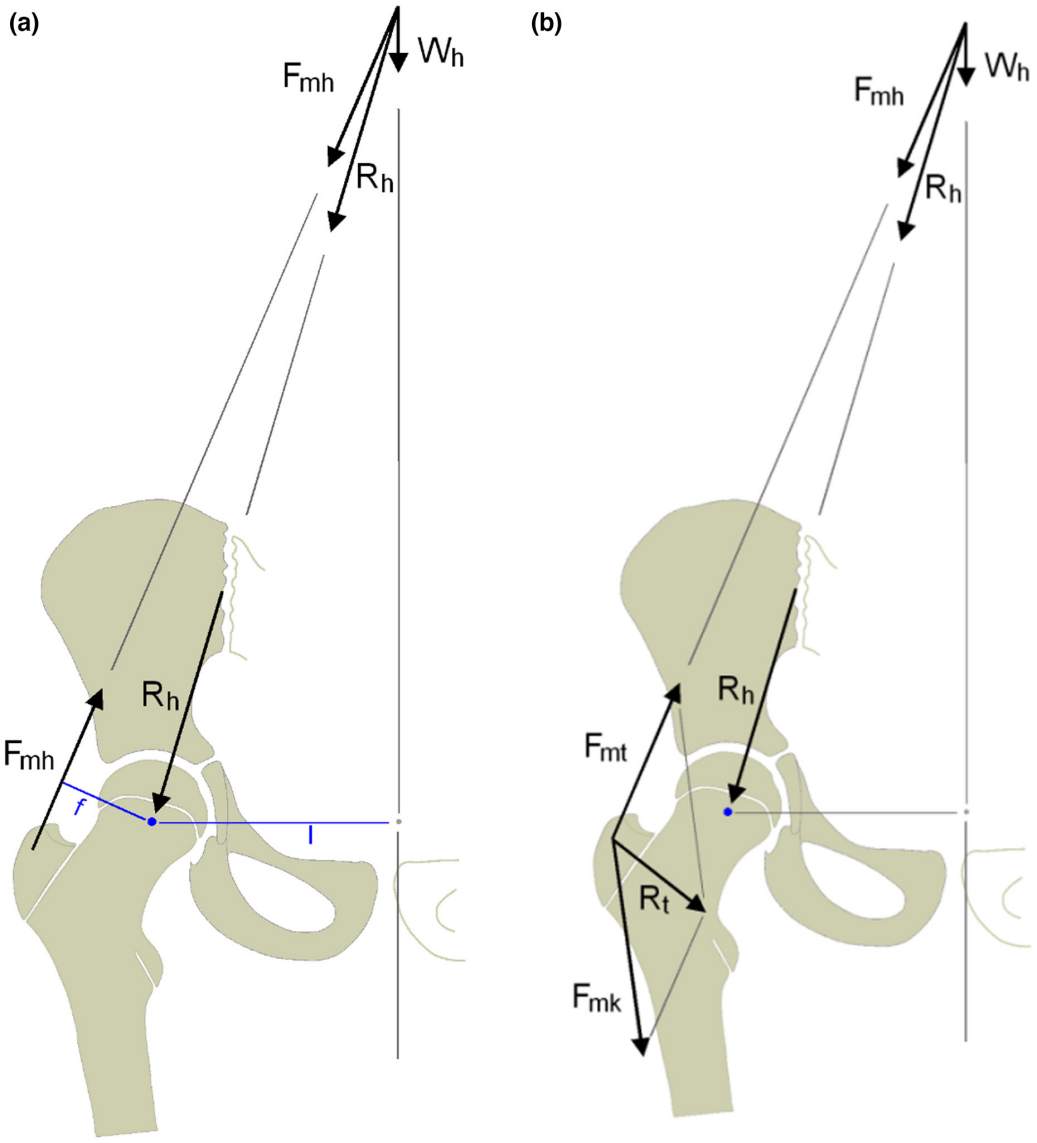
(a and b) Comparison of the classic biomechanical hip model as described by Pauwels (1a, from Engelhardt & Raschke, 2020) and the refined model by Heimkes (1b, from Skuban et al., 2009), that accounts for the regional force vectors located at the trochanteric region. *F*_mh_, Force vector of the abductor muscles; *F*_mk_, Force vector of the knee extensors and the iliotibial band; *F*_mt_, Force vector of the abductors; *R*_h_, Resulting force vector of the hip; *R*_t_, Resulting force vector acting on the greater trochanter; *W*_h_, body weight without the leg in stance.

Apophyseal growth plates, unlike epiphyseal growth plates, do not contribute to the longitudinal growth of the skeleton. However, they have a decisive influence on the development, shape, and structure of the associated joint (Heimkes, 2016). Growth plates generally align perpendicular to the forces acting on them, as described by Pauwels, Carter, Frost, and Milz (Carter et al., 1987; Frost, 1994; Milz et al., 2002; Pauwels, 1948, 1965), resulting in a flat or slightly concave shape. Morscher (1961) reports convex shapes or a bend at the edges that serves as resistance to transverse shear forces. Putz (1996) describes grooves and ridges at the interface to minimize transverse shear. To our knowledge, only Heimkes (2016) and Heimkes et al. (1993) have reported central, nonmarginal twisting of a growth plate. In the human skeleton, there are three curved or distorted growth plates, one of which is the proximal growth plate of the tibia, which curves almost perpendicularly due to the influence of the resultant force vector of the knee joint on the one hand and the additional effect of the patellar tendon on the tibial apophysis on the other. The second is the growth plate of the proximal humerus, which has an angled shape during growth. The third is the growth plate of the greater trochanter. As described by Heimkes, its ventral and medial parts are oriented mainly parallel to the femoral neck. According to Pauwels, this is the result of a distally occurring reorientation of the resultant force vector of the hip (introduced by him as R), due to a tension band effect caused by the iliotibial band (Eschweiler et al., 2010; Pauwels, 1965). Heimkes et al. (1993) refined and concretized this concept by introducing the resulting trochanteric force vector Rt as the product of the force *F*_mt_ of the abducting muscles (glut. med., min., max. and tens. fasc. lat.) and Fmk of the knee extensors (without rect. fem.) and the iliotibial band (Figure 1).

**FIGURE 2:**
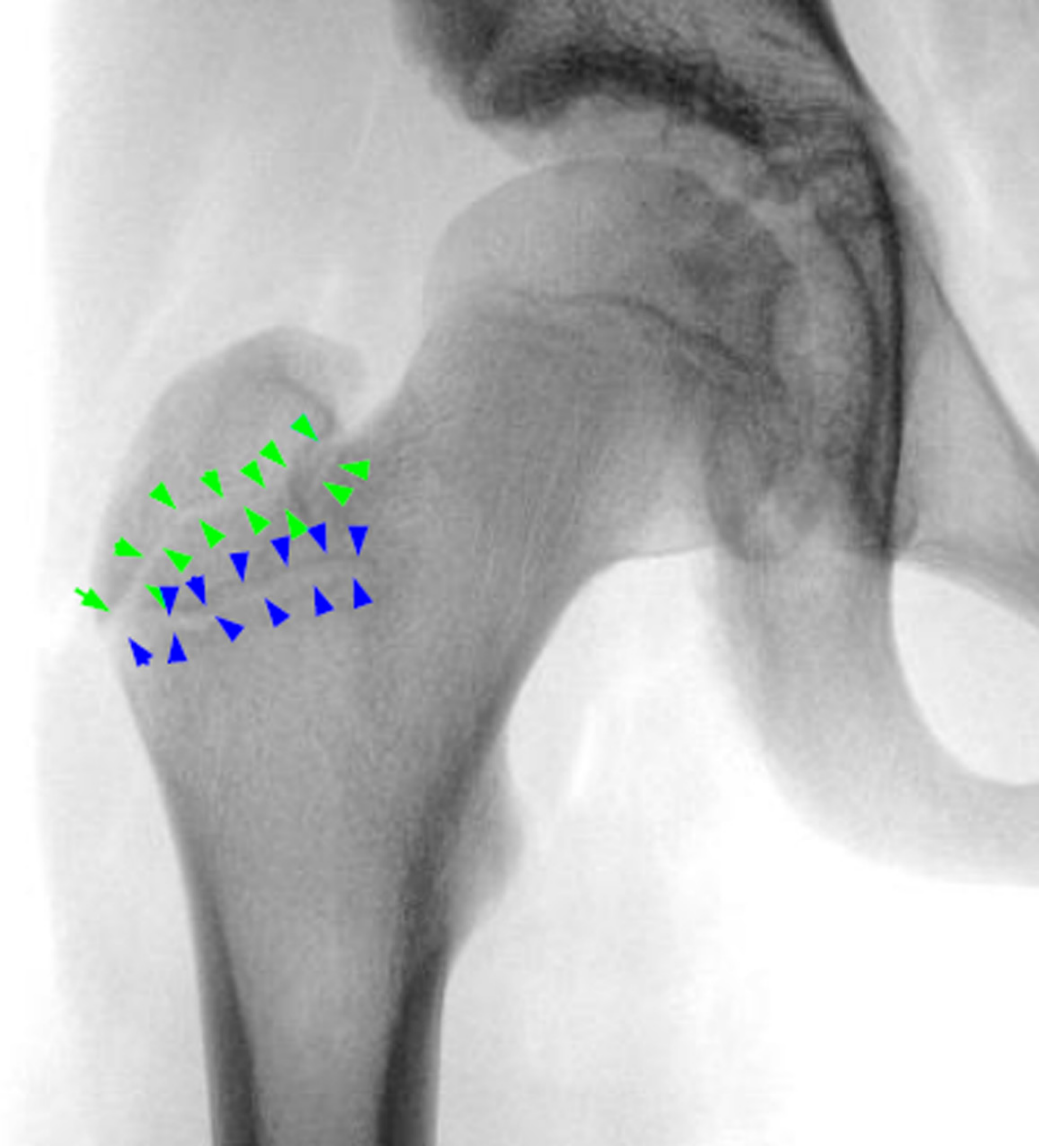
Conventional radiograph of a skeletally immature right proximal femur. The outlines of the ventral side of the greater trochanteric apophysis are marked with green arrows, the dorsal side with blue arrows.

**FIGURE 3:**
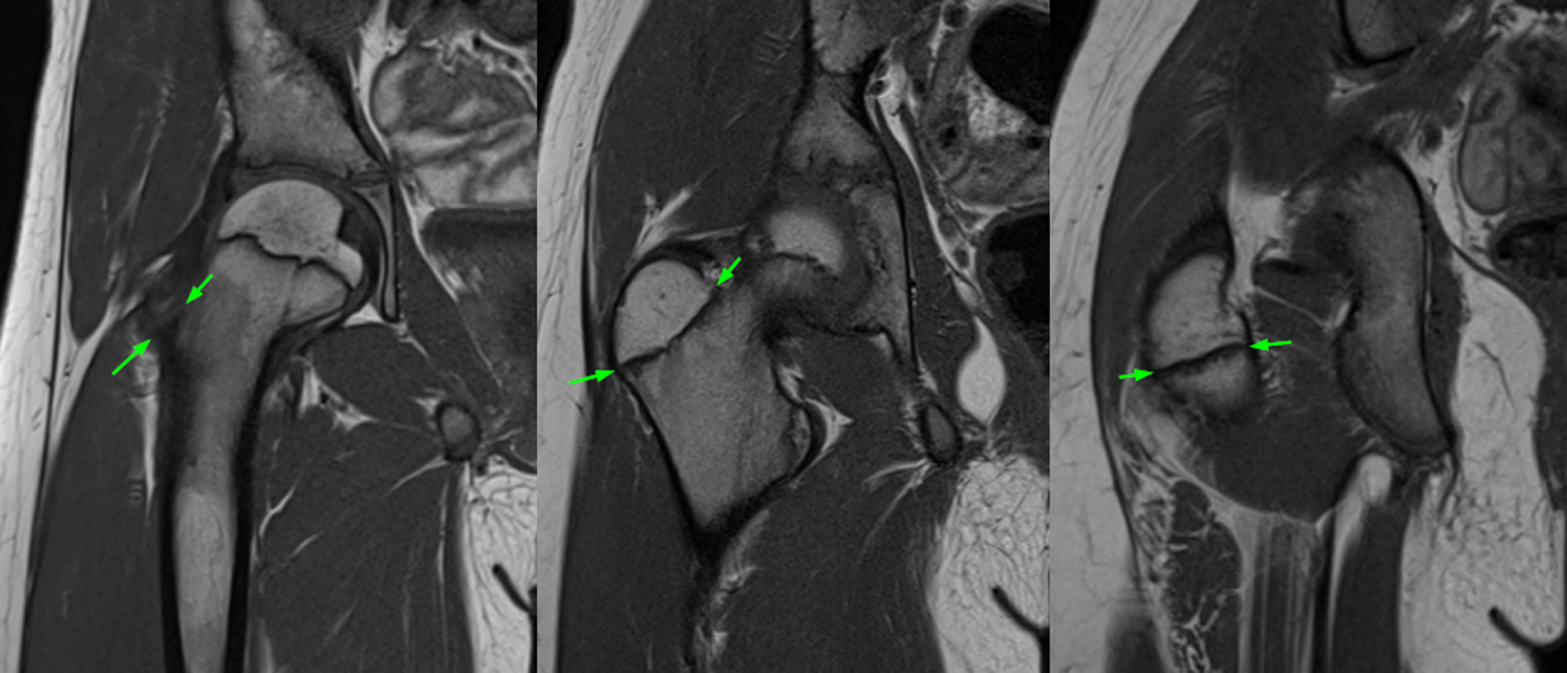
Magnetic resonance imaging, T1 sequence, of the right hip of a 10‐year‐old girl. The arrows mark the lateral and medial margins of the apophyseal plate. In the ventral and medial part of the apophysis (left and middle images), the apophyseal plate is aligned parallel to the femoral neck. The image on the right shows the dorsal aspect of the apophysis with a more horizontally oriented growth plate.

Looking at a conventional radiograph of a neutrally rotated proximal femur of a skeletally immature individual, two areas with a double border can be seen (Figure 2). The more proximal structure (green arrows) is aligned along the femoral neck axis and represents the ventral aspect of the growth plate. Comparing the images with the corresponding MRI images (Figure 3), it can be seen that the more distal and more horizontally oriented structure (blue arrows) represents the dorsal part of the growth plate.

In the absence of detailed measurements, our first step was to generate geometric data on the horizontal reorientation of the growth plate from anterior to posterior. While current biomechanical models account for the orientation of the ventral portion of the growth plate, they do not account for the apparent deviation in orientation of the dorsal aspect of the growth plate. Therefore, second, we hypothesized that the dorsal part is subjected to different forces generated by the external rotators.

## 2 METHODS

We retrospectively analyzed 57 anonymized magnetic resonance imaging images (MRI) of different patients who had previously been admitted to the radiology department of the LMU University Hospital for various reasons. Fifty‐one of the patients were between 4 and 17 years of age while six patients were between 18 and 52 years of age. Only MRIs without pathologic findings were considered. Specifically, we excluded scans with pathologic neck‐shaft angles, congenital deformities, and patients who had previously undergone surgery. According to our internal protocol, patients were positioned in a standardized manner with legs turned slightly inward. Images of patients who were not ideally positioned were excluded. We used Osirix (Pixmeo SARL) to analyze the DICOM images.

To compare apophyseal plate measurements, we defined a calibration slice to account for differences in patient size, volume, and slice thickness. This layer was set to the maximum longitudinal extent of the apophyseal growth plate in each patient. The calibration layer is marked in red in the diagrams and labeled “6”. Five slices ventral and dorsal to the calibration slice were then recorded and labeled and measured accordingly. To determine the vertical axis of the body in a dataset, a dorsal layer is used with the center of the sacral vertebrae and the center of the anus as reference points. The orthogonal line to this axis is the horizontal axis, which is then projected onto the other slices. The angle between the apophyseal growth plate and the horizontal plane gives the AY angle of each frontal layer.

For axial calibration, the layer with the largest diameter of the greater trochanter was used. On average, the greater trochanter is still visible in four slices cranial and four slices caudal to this slice in the MRI images. Thus, layer five serves as the reference layer in the axial MRI image studies. For layers “x” cranial to the reference layer, the function f(x) = 5−x; for “y” layers caudal to the reference layer, the function f(y) = 5 + y.

**FIGURE 4:**
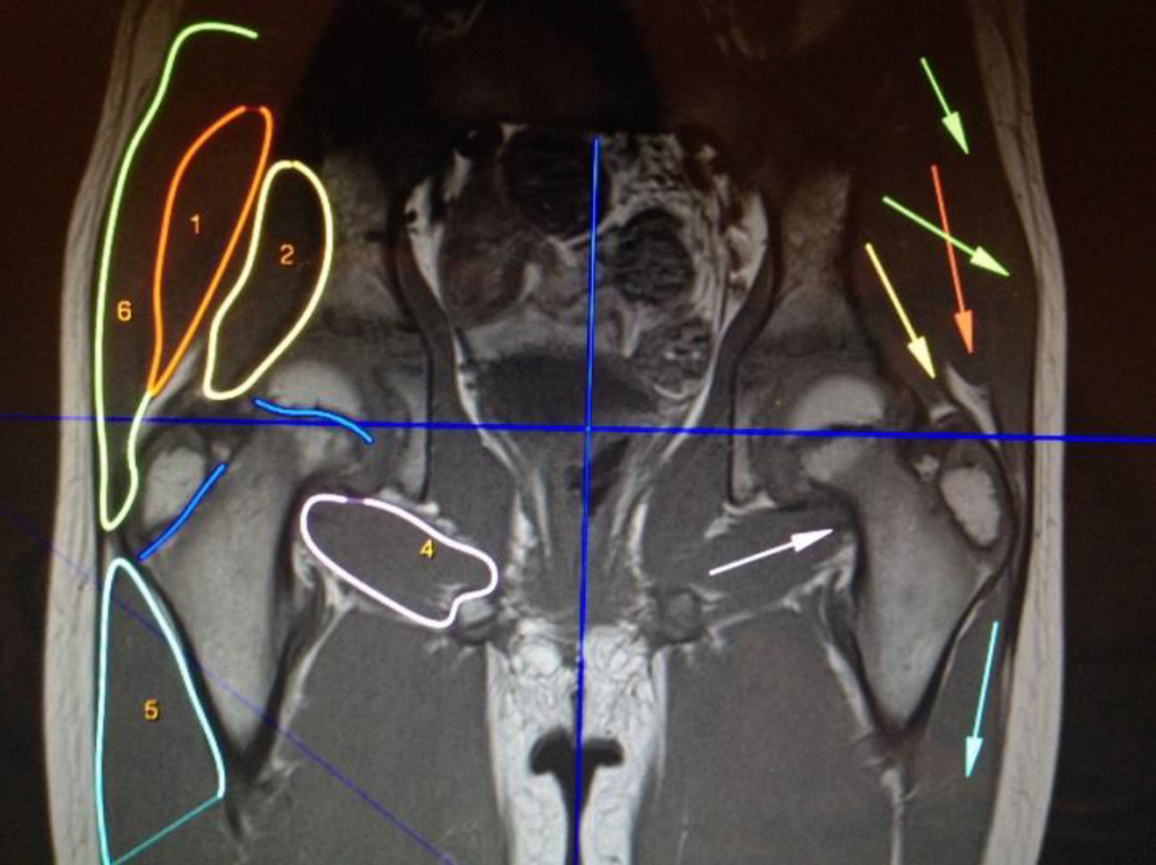
Frontal T1‐MRI with outlines (patient right) and straight lines (patient left). 1 Gluteus medius; 2 gluteus minimus; 4 quadratus femoris; 5 vastus lateralis; 6 gluteus maximus.

**FIGURE 5:**
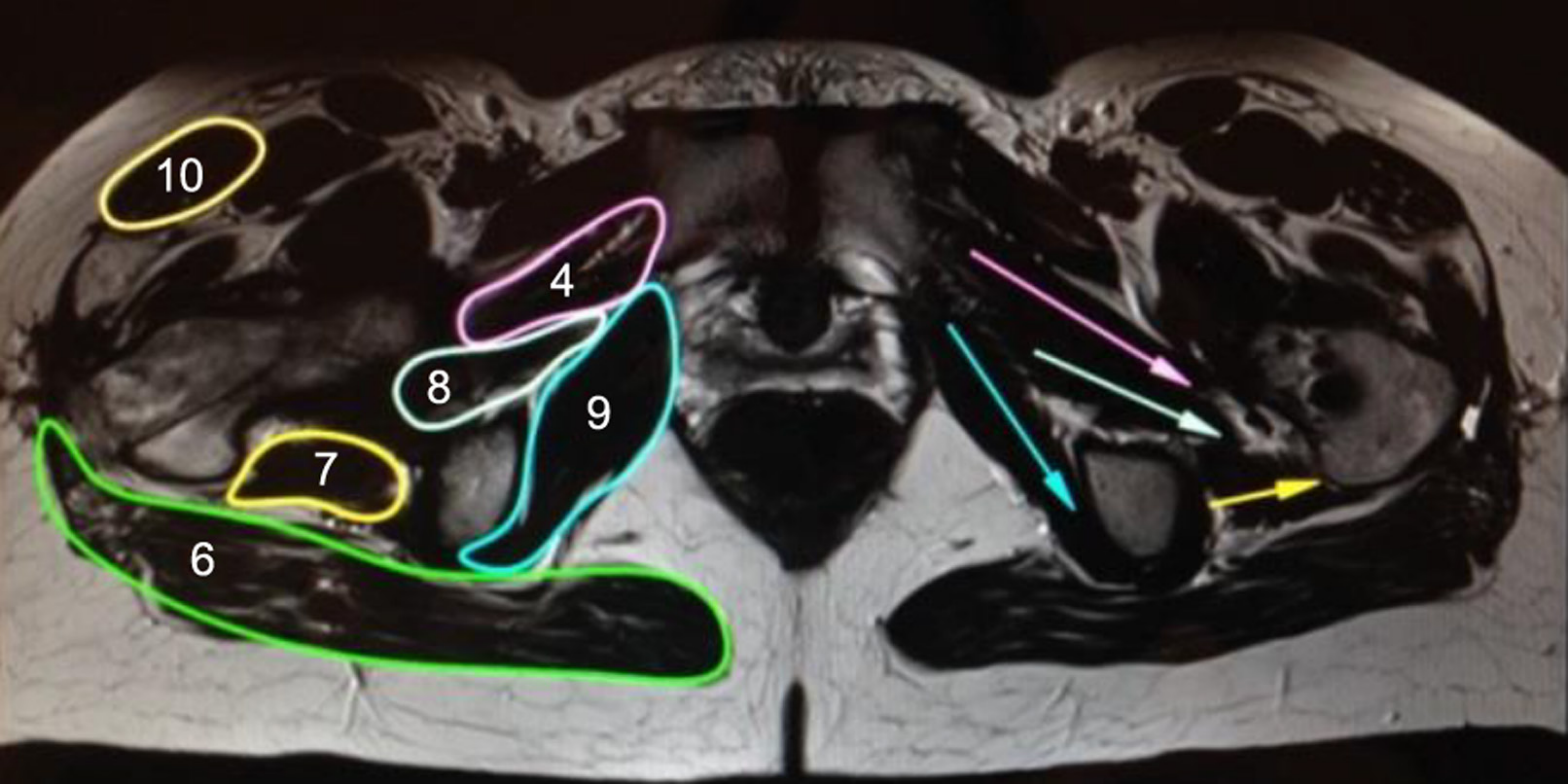
Axial T1‐MRI scan with outlines (patient right) and straight line markups (patient left). 4 Triceps coxae; 6 gluteus maximus; 7 quadratus femoris; 8 piriformis; 9 obturator internus before deflection; 10 tensor fasciae latae.

To visually infer the effects of muscle strength, we used rectilinear models (Dostal & Andrews, 1981). First, we isolated individual muscles by outlining their boundaries to delineate them from surrounding structures and clearly identify their points of origin and attachment. Using the magnification function of Osirix, the direction of muscle fibers can be determined and marked with arrows (Figures 4 and 5). If the fibers differ in direction, more attention is paid to the fibers located centrally in the muscle. For muscles with regionally different muscle fiber orientations, such as the gluteus maximus muscle, the different regions are marked separately. In the frontal view, for example, the cranial and caudal portions of the gluteus maximus muscle are distinguished.

## 3 RESULTS

### 3.1 AY‐angle

**FIGURE 6:**
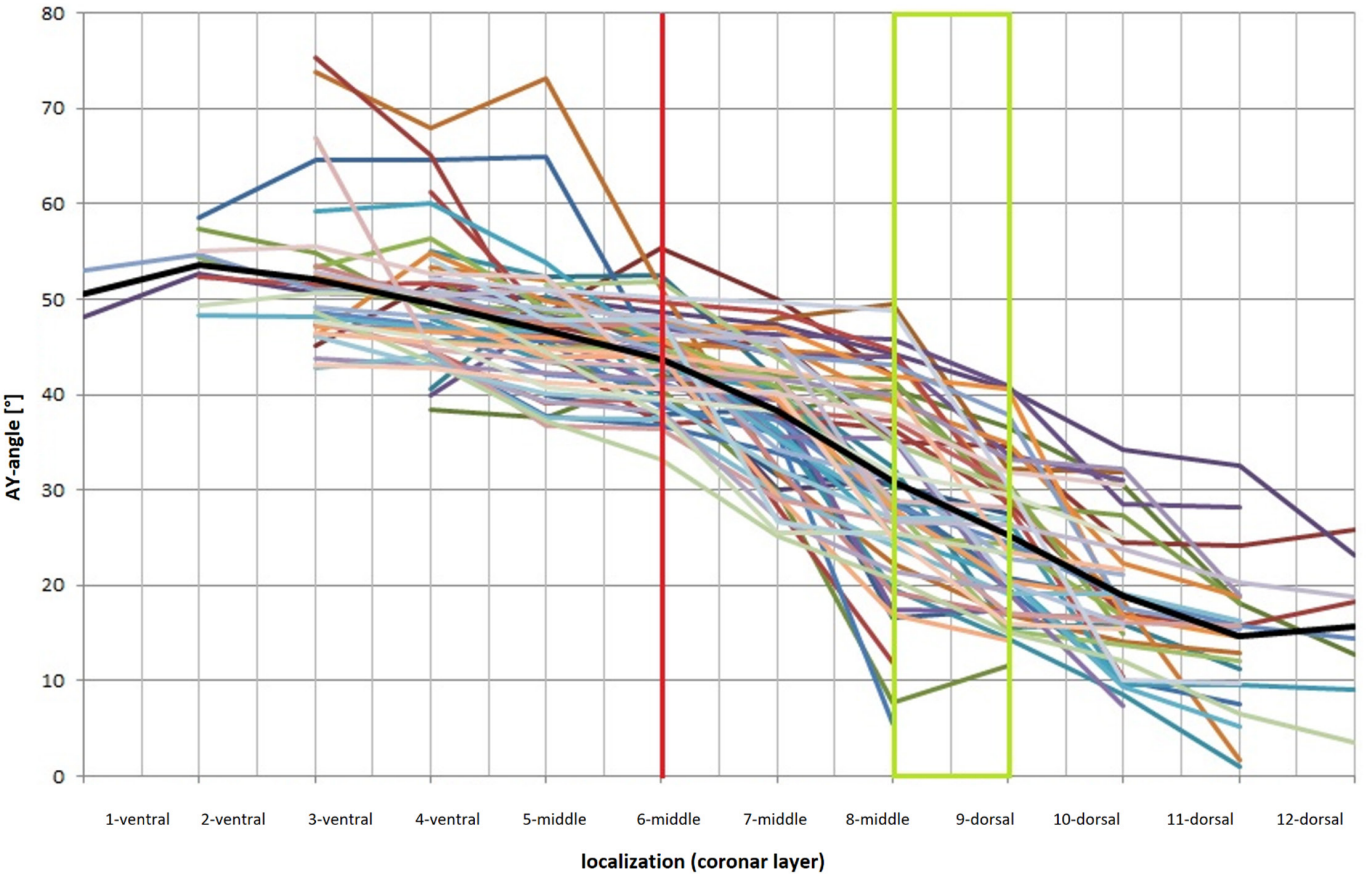
The inclination of the apophyseal plate with respect to the body horizontal (= AY angle) for 12 layers, from ventral (left) to dorsal (right). The black line represents the mean value, the red line represents the layer with the maximum length of the apophyseal plate and the green box represents the transitional area between the middle and dorsal thirds of the apophyseal plate.

**FIGURE 7:**
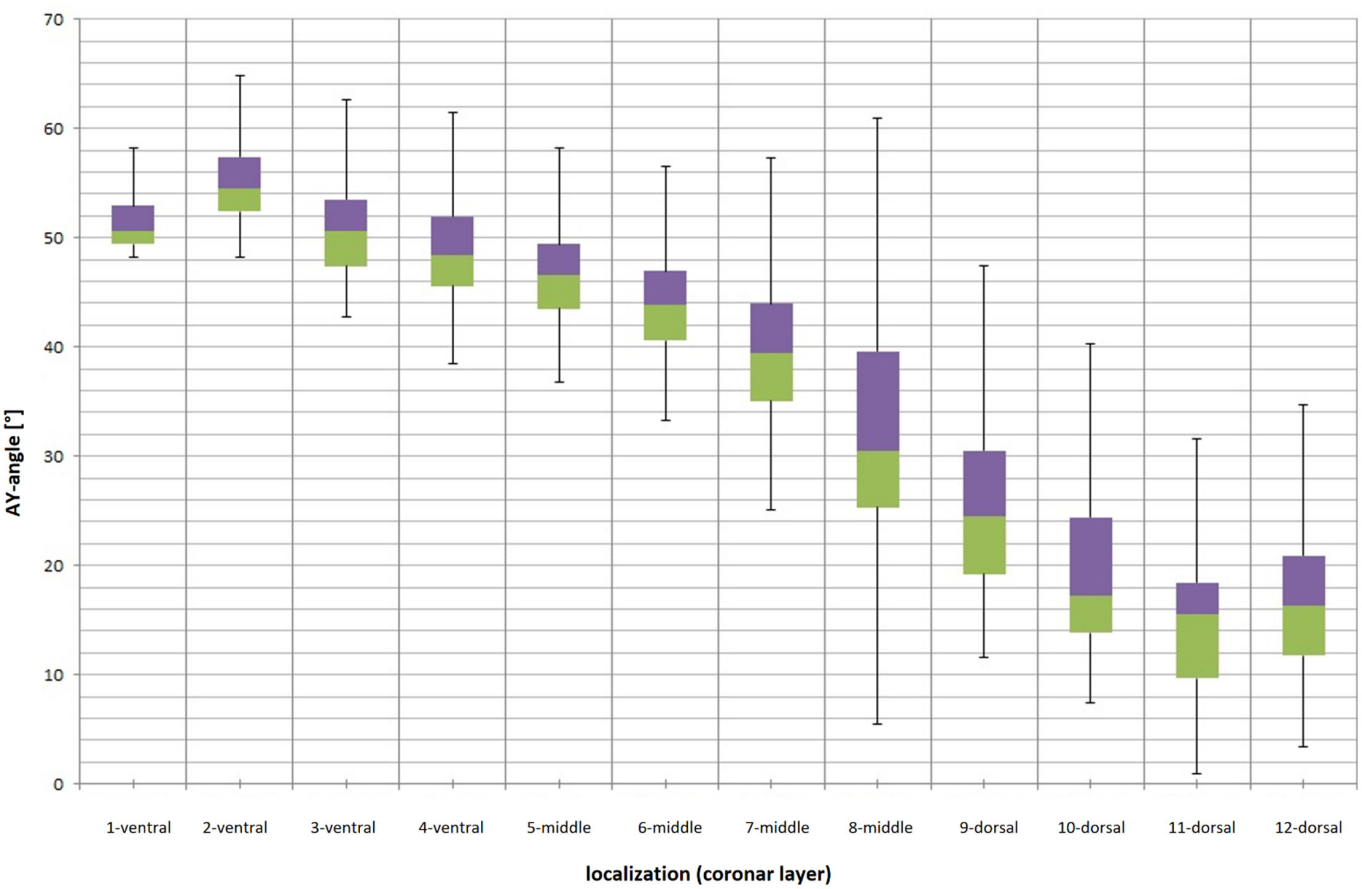
Inclination of the apophyseal growth plate with respect to the body horizontal (= AY angle) in the coronal plane, in 12 layers, stratified from ventral to dorsal. Fifty‐seven hips of various ages were measured.

All 57 patients we examined showed a significant decrease in ventrodorsal AY angle of 33.44° on average. Between the first and second layers, the AY angle increases slightly by two degrees, followed by a decrease until the eighth layer. Subsequently, we observed a jump in the AY angle between layers eight and nine, which is marked as the transition zone in the summary plots (Figures 6 and 7). From layer nine, the AY angle becomes more stable. Interestingly, the transition zone is located between the middle and dorsal thirds of the apophyseal plate. In layer six, which is the calibration layer with the greatest length of the apophyseal plate, the growth plate is at an angle of 43.7° to the horizontal plane. In the ventral third of the apophyseal plate, the AY angle averages 51.64°, while in the dorsal third, it is 18.6°.

**FIGURE 8:**
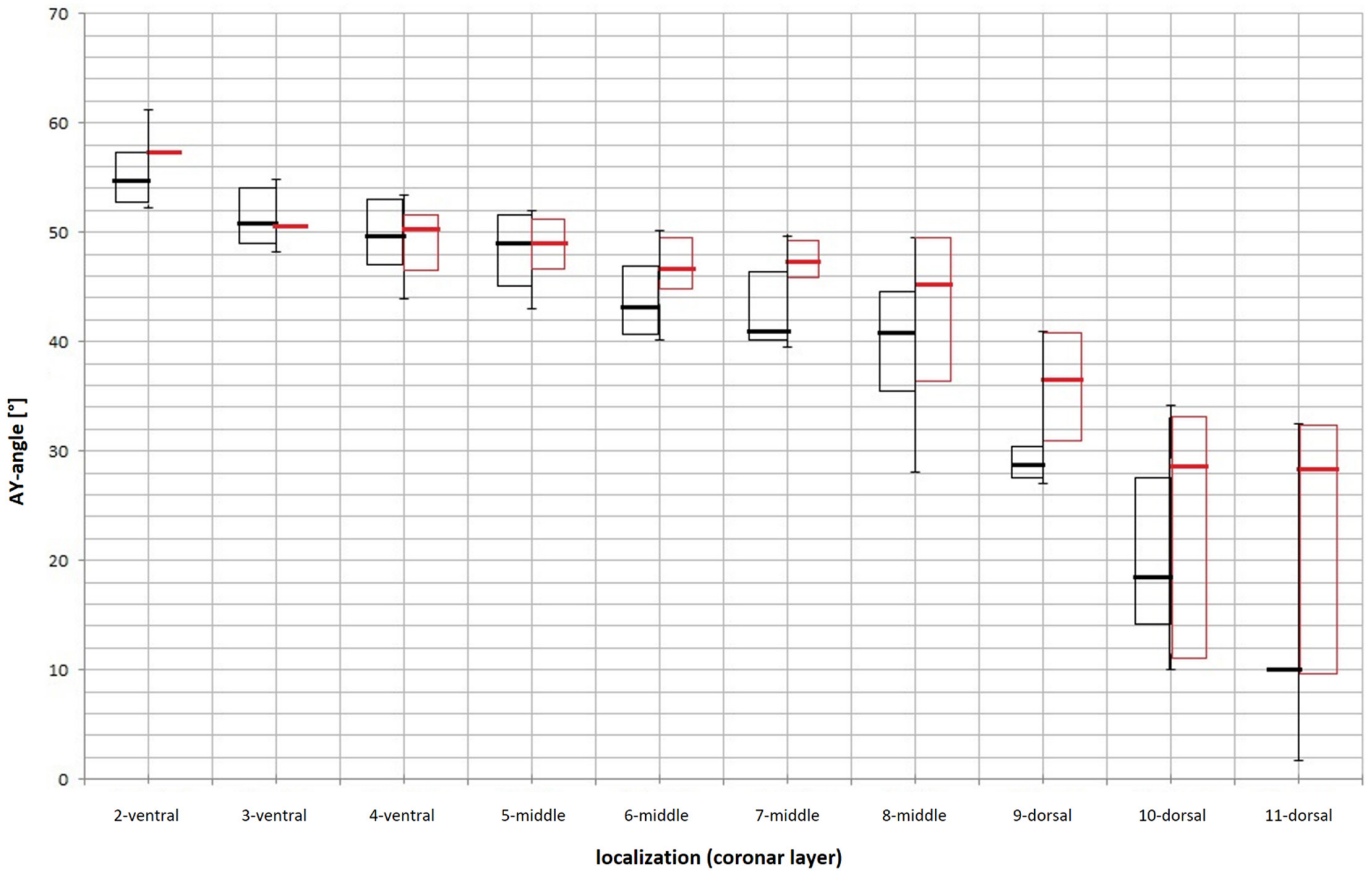
Boxplots of the AY‐angles of five pediatric hips (red) and five adult hips (black). The pediatric hips clearly show less decline in AY‐angle toward the dorsal third of the apophysis.

**FIGURE 9:**
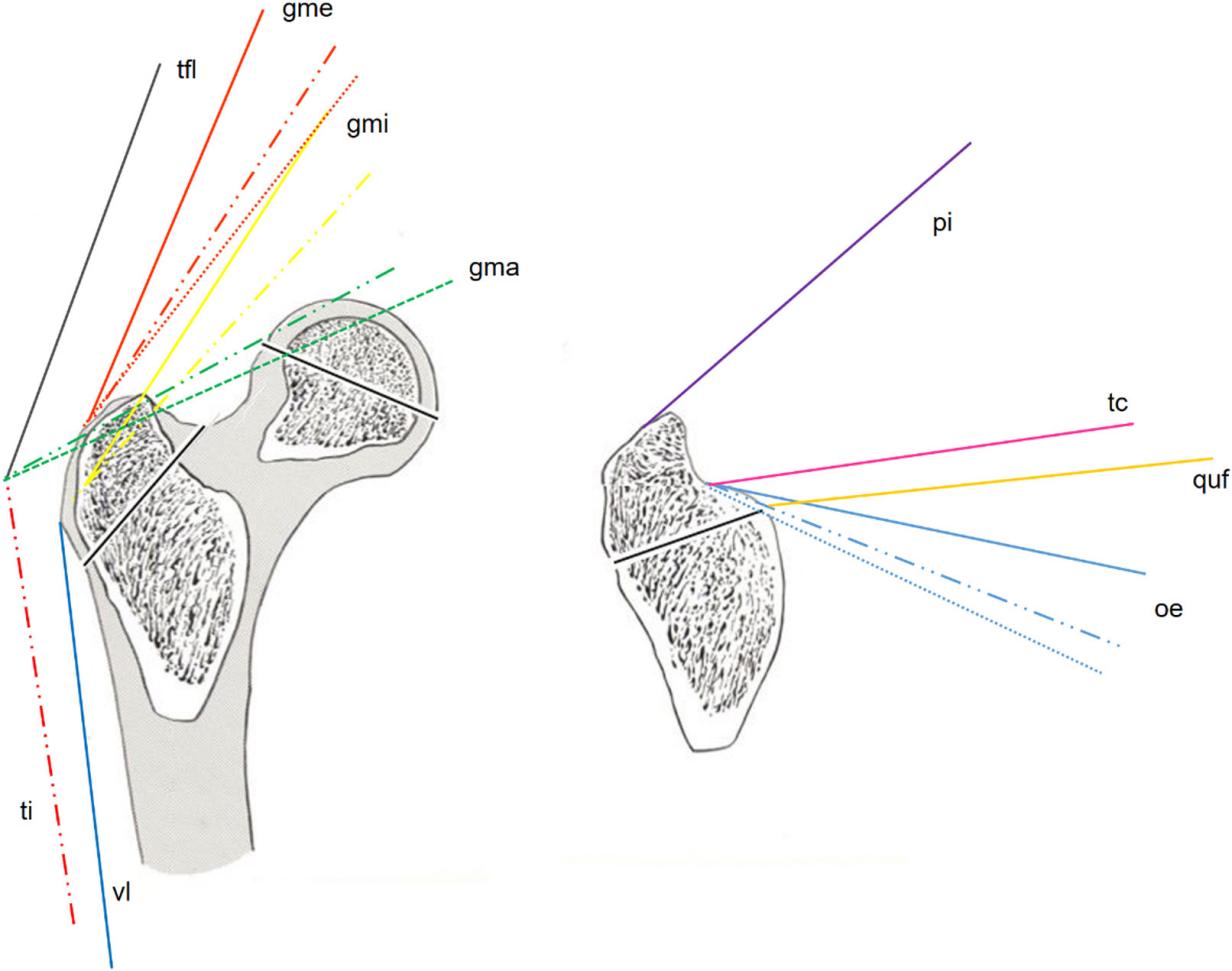
Schematic of the lateral internal and external as well as the posterior tension band systems at the trochanter major in the frontal plane. (a) Ventral and median layers: gma, gluteus maximus; gme, gluteus medius; gmi, gluteus minimus; tfl, tensor fasciae latae; ti, iliotibial tract; vl, vastus lateralis; (b) dorsal layers: ge, gemelli; oe, obturator externus; pi, piriformis; quf, quadratus femoris; tc, triceps coxae. The continuity of the lines represents the localization: continuous line: mean; for lines in the same colour: continuous line ventral third; line/dot/dot/line median third; dotted line dorsal third.

**FIGURE 10:**
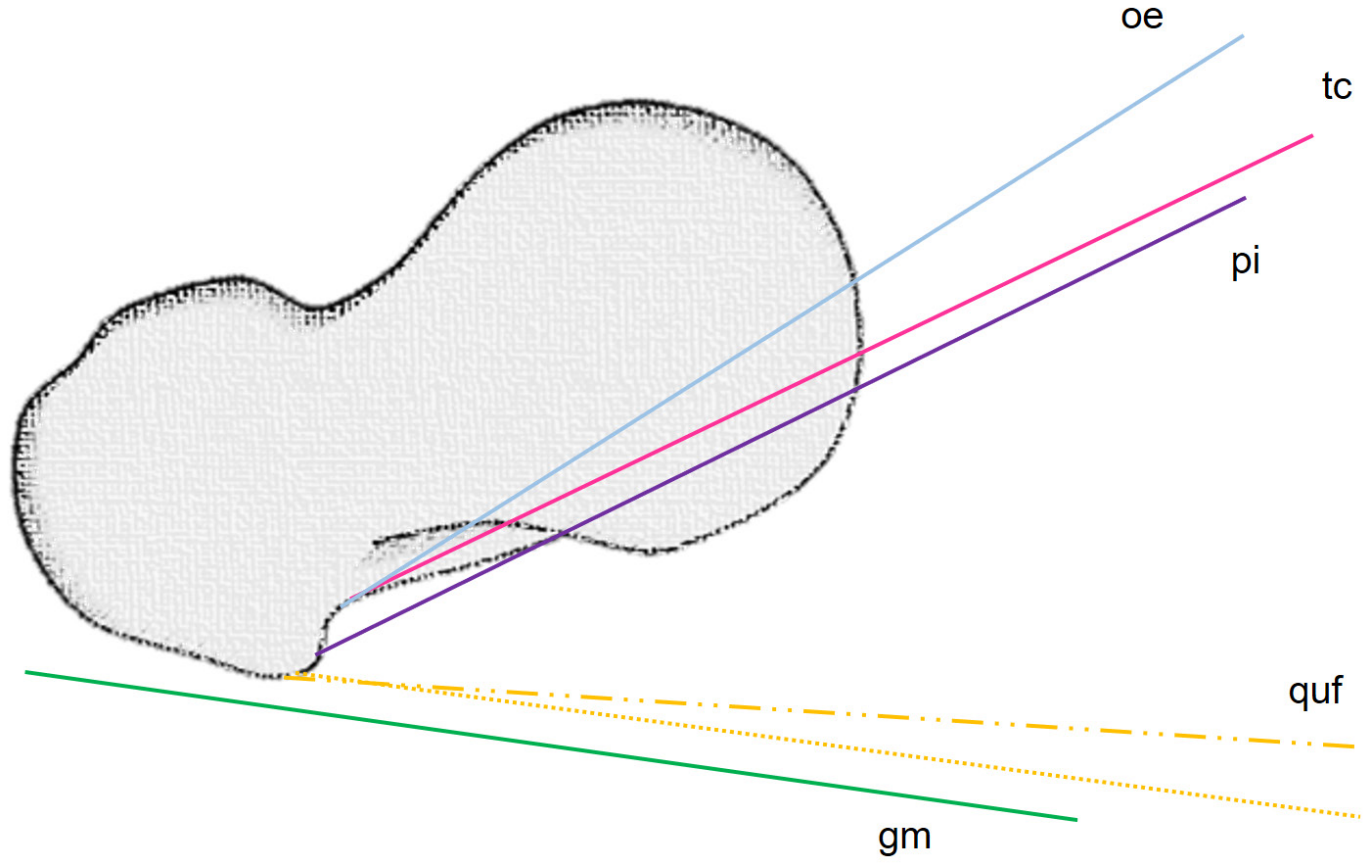
Schematic of the external and posterior tension band systems at the trochanter major in the transverse plane. gm, gluteus maximus; oe, obturator externus; pi, piriformis; quf, quadratus femoris; tc, triceps coxae.

Comparing pediatric hips and adult hips randomly selected from both age groups, the pediatric hips showed a smaller decrease in AY angulation toward the dorsal edge of the apophysis (Figures 8, 9, 10).

### 3.2 Muscle trajectories in frontal plane

Frontal measurements of the muscle fibers show that only the gluteal muscles change their orientation to the horizontal axis and approach it in the dorsal direction (Figure 9). The greatest angular change is observed in the gluteus medius, with a greater difference from ventral to dorsal in juvenile hips than in adult hips. In addition, in frontal view, several muscles, including gluteus medius, quadratus femoris, obturator externus, and triceps coxae, are more steeply oriented in juvenile hips than in adult hips (Table 1).

**TABLE 1:** Direction of progression of the individual muscle fibers in the frontal plane in relation to the horizontal.

Trajectory (frontal plane)	Color	Localization	Mean [°]	Mean [°]	Mean [°]
			*n* = 10 (total)	*n* = 5 (adults)	*n* = 5 (children)
Gluteus maximus (caudal)		Ventral			
		Middle	62.46	65.2	60.62
		Dorsal	66.33	68.72	64.38
Gluteus maximus (cranial)		Ventral			
		Middle	50.72	52.52	49.4
		Dorsal	52.62	51.95	53.43
Iliotibial band		Ventral			
		Middle	6.48		
		Dorsal	8.53		
Tensor fasciae latae		Mean	19.85	20.14	19.35
Gluteus minimus		Ventral	32.99	34.49	31.76
		Middle	43.36	44.17	42.42
		Dorsal			
Gluteus medius		Ventral	22.55	26.44	19.57
		Middle	33.56	37.01	30.29
		Dorsal	38.08	39.21	34.68
Vastus lateralis		Ventral	−3.9	−3.9	
		Middle	−6.35	−5.46	−7.21
		Dorsal	−8.24	−8.39	−8.08
Piriformis		Ventral			
		Middle	48.64	47.12	49.65
		Dorsal	49.2	48.18	50.74
Triceps coxae		Ventral	80.43	84	78.64
		Middle	82.29	84.19	79.19
		Dorsal			
Obturatorius externus		Ventral	−77.9	−79.66	−78.51
		Middle	−69.06	−70.34	−68.03
		Dorsal	−65.4	−67.3	−62.21
Quadratus femoris		Ventral			
		Middle	82.21	85.64	76.74
		Dorsal	82.72	86.62	80.12

*Note*: Subdivision according to ventral, midline, and dorsal localization (thirds of the layers) as well as according to patient group (total, adult, and juvenile).

### 3.3 Muscle trajectories in transverse plane

In axial view, the quadratus femoris muscle is the only muscle that undergoes a relevant angular change from frontal to caudal (Figure 10). In addition, differences between juvenile and adult muscles can be observed in the quadratus femoris muscle, with the muscle trajectory steeper by an average of four degrees in children. All muscles attaching to the medial aspect of the greater trochanter have a nearly parallel orientation from dorsolateral to centroventral (Table 2).

**TABLE 2:** Direction of progression of individual muscle fibers in the transverse plane in relation to frontal.

Trajectory (transverse plane)	Color	Layer	Mean [°]	Mean [°]	Mean [°]
			*n* = 10 (total)	*n* = 5 (adults)	*n* = 3 (children)
Gluteus maximus		Cranial	−8.56	−8.61	−8.49
		Middle	−8.34	−8.41	−8.23
		Caudal	−8.33	−8.79	−7.61
Piriformis		Cranial	22.38	22.4	22.33
		Middle	22.37	22.3	22.49
		Caudal	22.39	22.36	22.41
Triceps coxae		Cranial			
		Middle	26.95	27	26.91
		Caudal	25.16	23.84	26.91
Obturatorius externus		Cranial			
		Middle	32.64	30.19	35.08
		Caudal	31.04	29.16	34.42
Quadratus femoris		Cranial			
		Middle	−8.09	−7	−10.56
		Caudal	−4.86	−4.77	−5.12

*Note*: Subdivision according to ventral, midline, and dorsal localization (thirds of the layers) as well as according to patient group (total, adult, and juvenile).

## 4 DISCUSSION

### 4.1 Discussion of the method

This work relies on MRI datasets for analysis, as Lube has identified in vivo measurements on MRIs as the most reproducible method for determining anatomical structures (Lube et al., 2017). Studies show that there is a large overlap between magnetic resonance measurements and anatomical cross‐sections (Engstrom et al., 1991). There are two different methods to calculate the direction of a muscle's course. The first is the centroid method, in which the midpoints of each muscle cross‐section are connected (Jensen & Davy, 1975). The second is the straight‐line method, which we used in this study and in which the origin and attachment points of the muscle are connected with a line (Dostal et al., 1986). The centroid line method has a higher potential for error because of the large number of measurements required, especially for muscles with different degrees of pinnation (Dostal & Andrews, 1981). In the transverse plane, the dissection method has a significant advantage over analysis of MRI datasets. Muscles that are nearly perpendicular to the cross‐sectional image, such as the gluteus medius, cannot be accurately determined with MRI datasets because the fibers are dissected.

### 4.2 Discussion of the results

#### 4.2.1 Muscle trajectories

To the best of our knowledge, this paper is the first to provide an overview of the trajectories of all muscle groups that attach to the greater trochanter (Tables 1 and 2). In 2010, Neumann (2010) examined the course of the hip muscles in detail, but did not address their attachments and considered the hip joint as a whole. He also did not address whether the individual muscles work together to form muscle loops or tension bands. Heimkes focused mainly on the interaction of the individual muscles (Heimkes et al., 1992, 1993). He described the muscle insertions at the apophysis of the great trochanter as well as the vastogluteal muscle sling (internal tension band) and the external tension band at the greater trochanter, but without considering the external rotators. He described the torsion but could not provide an explanation for it.

Both Dostal and Dunkelberg calculated the anatomical course of the hip muscles by analyzing anatomical specimens that they mounted on a gauge while maintaining equilibrium according to Newton's law (Dostal & Andrews, 1981; Dunkelberg, 1989). Dunkelberg used a preparation method in which he determined the muscle origin and attachment points using a coordinate system. He calculated the angle to the vertical as follows: = − arc tan (d/Y(R)), where “d” is the lever arm length and “Y(R)” is the vector of the y coordinate. Neumann illustrated the values obtained by Dostal for the muscle courses. Like Seireg and Arvikar (Seireg & Arvikar, 1973) and the authors of this paper, he used the straight‐line method. Jensen also used cadaver specimens but visualized the trajectories using the centroid line technique (Jensen & Davy, 1975; Jensen & Metcalf, 1975).

Compared to their data, we found higher angular values for the frontal plane (Tables 3, 4, 5). This could be due to the fact that the gluteal muscles are composed of several parts, and it is not known whether they were differentiated in these studies. The orientation of the gluteal muscles changes along their course. In images that are far ventral, smaller angles to the vertical are observed. It is possible that both Dunkelberg and Neumann measured the muscle courses only ventrally. An indication for this hypothesis would be that the angle values we measured for the external rotators do not show any changes from ventral to dorsal and thus agree well with the data from the literature.

**TABLE 3:** Comparison of muscle trajectories on images in the frontal plane in relation to the body horizontal (the minus sign indicates a caudomedial trajectory).

	Gluteus maximus [°]	Tensor fasciae latae [°]	Gluteus minimus [°]	Gluteus medius [°]
Dostal/Neumann	30	4	24	22.5
Dunkelberg	44.6375	7.45	28.725	12.14166667
Seireg, Arvikar				30.33
Jensen, Davy				41.3
This work	58.0325	19.85	38.175	31.4

**TABLE 4:** Comparison of muscle trajectories on images in the frontal plane in relation to the body horizontal (the minus sign indicates a caudomedial trajectory).

	Piriformis	Triceps coxae	Obturatorius externus	Quadratus femoris
Dostal/Neumann	63°			−84.5°
Dunkelberg	66.425°	−78.14°	−73.55°	−73.55°
This work	48.92°	81.36°	−70.79°	82.47°

**TABLE 5:** Comparison of muscle trajectories on transverse slices in relation to the body vertical (minus sign indicates a dorsomedial course).

	Gluteus maximus	Tensor fasciae latae	Gluteus minimus	Gluteus medius
Dostal/Neumann	−39		−48.73	1.24
Dunkelberg	−28.86	4375	−61.19	−69.3
Seireg, Arvikar				−93.08
Jensen, Davy				−82.23
This work	−8.41			

Only the triceps coxae and quadratus femoris show a slightly more cranio‐medial course on frontal sections in this study (Table 4). This may be due to the fact that the majority of the hips measured in this study were from adolescent patients. Children typically have coxae valgae. As the CCD angle decreases, the relative position of the greater trochanter changes and the external rotators become even closer to horizontal or take a caudo‐medial course.

The trajectories in the transverse plane show comparable values for most of the muscles we examined (Table 6). However, we found a more parallel alignment to the frontal plane for the gluteus maximus. While the effect was especially pronounced in the immature population, our findings were consistent and constantly far less deviating from frontal than in the works of Dunkelberg and Dostal (Table 5). In the absence of alternative obvious explanations, we would assume an effect in the display of the gluteus maximus on the mrt and the subsequent transfer into trajectories.

**TABLE 6:** Comparison of muscle trajectories on transverse slices with respect to the body vertical (minus sign indicates a dorsomedial course).

	Piriformis	Triceps coxae	Obturatorius externus	Quadratus femoris
Dostal/Neumann	38	35.3	12	−6.8
Dunkelberg	24.55	38.58	18.62	−3
This work	22.38	26.06	31.84	−6.47

#### 4.2.2 The anterior and the posterior tension band system and the external rotators

**FIGURE 11:**
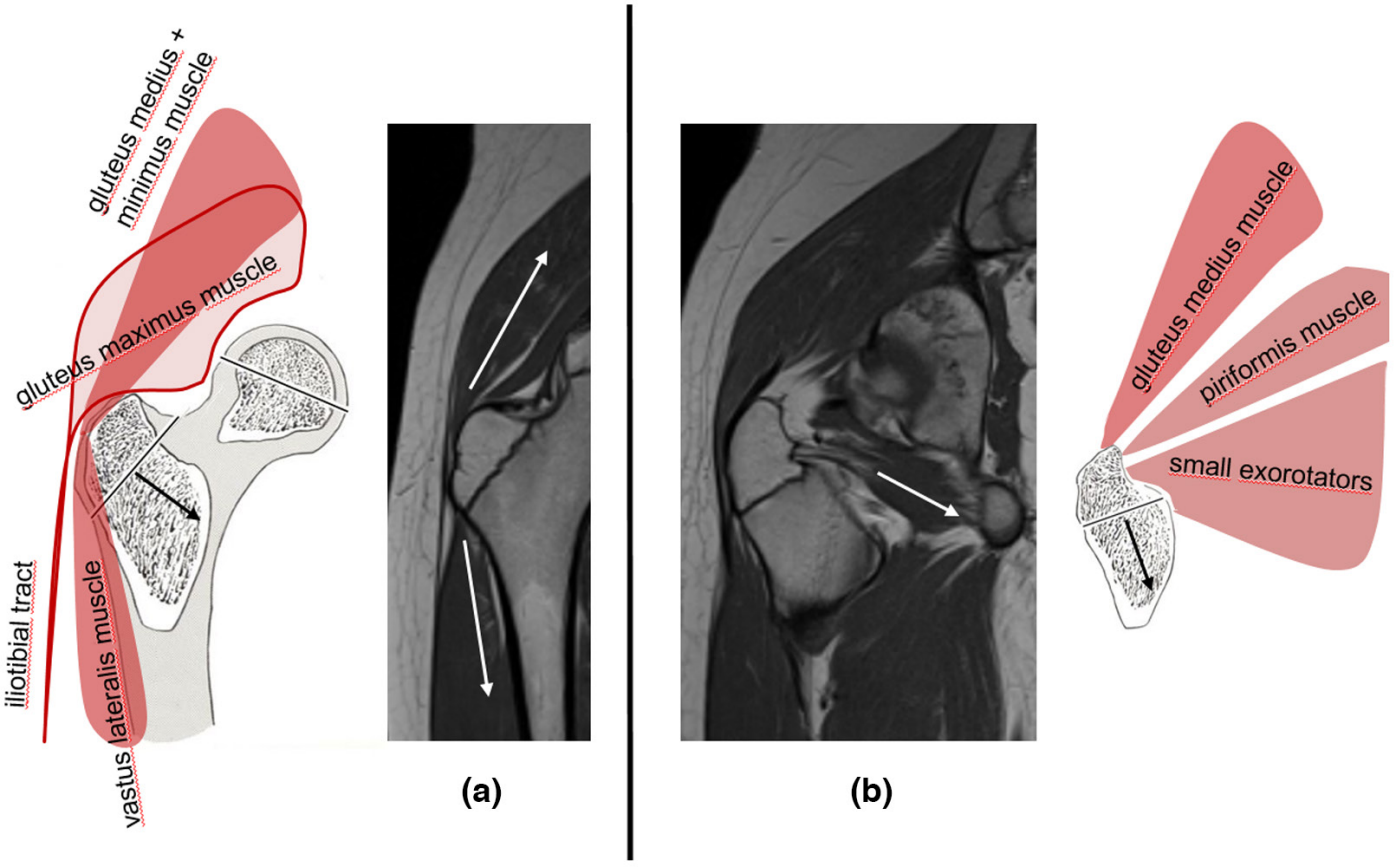
(a) The lateral tension band systems consisting of the external system (iliotibial band & gluteus maximus) and the internal system (vastus lateralis & gluteus medius and minimus); (b) The posterior tension band system (gluteus medius, piriformis and the small exorotators).

**FIGURE 12:**
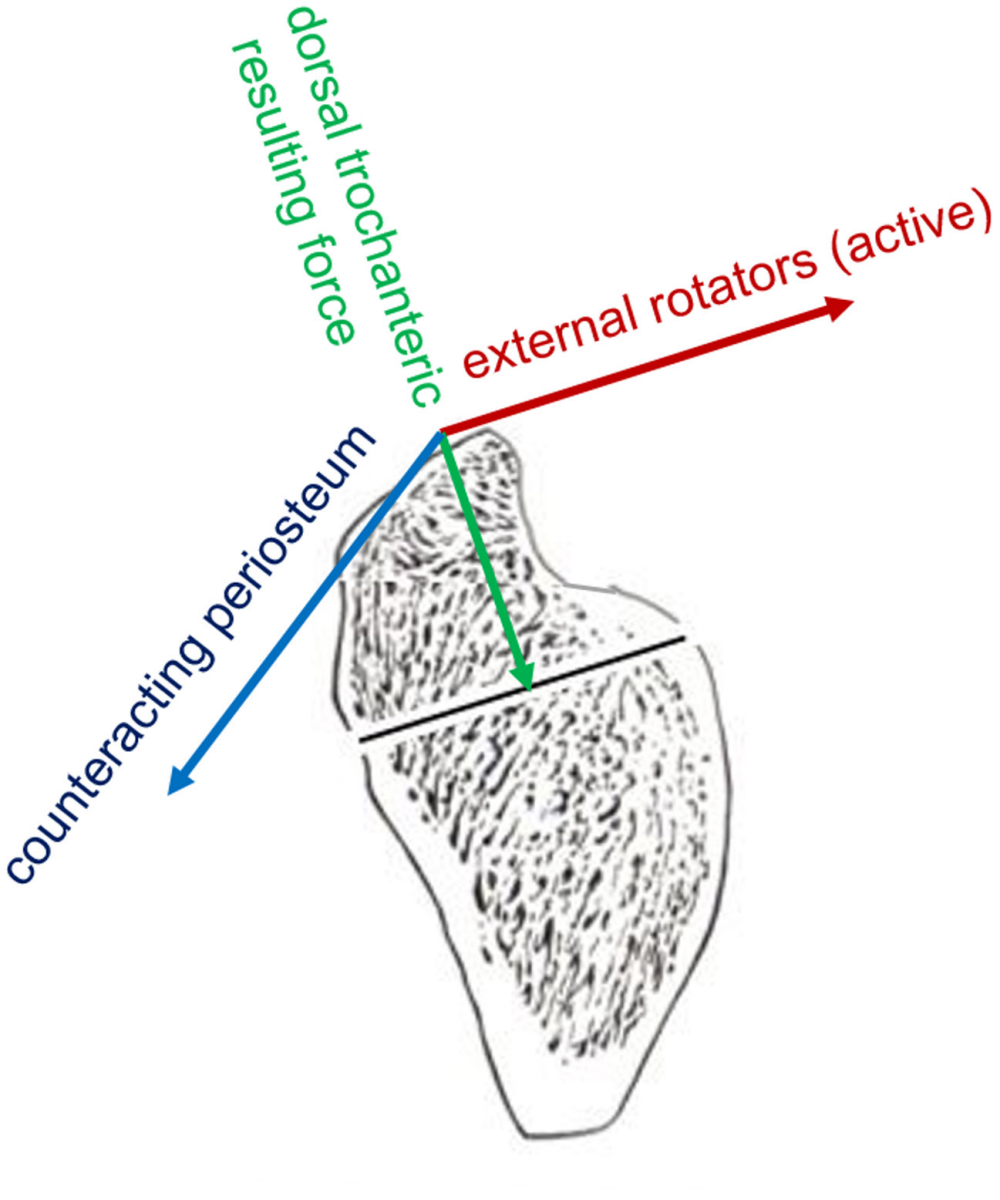
(a) Acting forces on the growth plate of the major trochanter; (b) Estimated parallelogram of the dorsal tension band system; green: resulting dorsal trochanteric force vector with almost vertical orientation, explaining the almost horizontal course of the dorsal part of the apophyseal plate; red: Force vector of the external rotators; blue: Counteracting force vector resulting from passive periosteal tension.

In summary, all measured muscle trajectories show that two tension band systems and an additional muscle force source in the form of the measured external rotators act on the trochanteric region (Figure 11). The internal tension band system is formed by the small gluteal muscles and vastus lateralis (as well as additional forces that are difficult to quantify), which result from fascial coupling of vastus intermedius and vastus medialis to vastus lateralis (Esterl, 1991; Heimkes, 2016). The external tension band is formed by the gluteus maximus, the tensor fasciae latae, and the iliotibial band. Both tension systems converge on the lateral aspect of the greater trochanter and exert pressure on it in a cranio‐lateral to caudo‐distal direction (Esterl, 1991; Heimkes et al., 1993; Figure 11a). The third source of force is represented by the external rotators (piriformis, triceps coxae, obturator internus and externus, and quadratus femoris), which, in contrast to the previously mentioned muscle groups, attach exclusively to the dorsal third of the trochanteric fossa (Figure 11b). The reason that the third system also acts like a tension band system is due to the fact that, although there are no muscle forces forming a muscle loop, the opposing force is created by a periosteal looping effect at the tip of the trochanter, which initially runs at a slight angle to the lateral side and then turns to the caudal side (Figure 12). This concept was previously presented by Putz and Milz (2016) as a way to neutralize forces in the absence of opposing muscle forces.

#### 4.2.3 The AY‐angle and its twist

According to previous data, the apophyseal growth plate at the greater trochanter is oriented at an angle of approximately 52° to the horizontal plane, which agrees well with the AY angles we found in the anterior two‐thirds of the growth plate. In the dorsal third, we observed a strong torsion with a flattening of the AY angle, most likely caused by the external rotator tension band system. Although the trajectories generated from our data are not suitable to explain the dorsal realignment of the growth plate when viewed in isolation, they do allow conclusions to be drawn when combined with the physiological cross‐sectional areas (PCSA) of the muscles available in the literature (Tables 7 and 8).

**TABLE 7:** Physiological cross‐sectional areas (PCSA) reported in the literature.

Muscle	PCSA [cm^2^]		
	Brand	Klein/Horsmann	Lube
Gluteus maximus	46.04	71.1	43
Tensor fasciae latae	5.23	8.8	5.9
Gluteus minimus	25.6	25.5	16.2
Gluteus medius	47.8	98.7	34.6
Vastus lateralis	40.445	34.85	42.6
Piriformis	14.85	8.1	11.2
Triceps coxae	4.63	33.6	21.7
Obturatorius externus	3795	24.6	8.2
Quadratus femoris		14.6	4.6

**TABLE 8:** Physiological cross‐sectional areas (PCSA) [cm^2^] reported in the literature, grouped by A: proximally directed muscles (glut. max., glut. med., glut. min., tens. fasc. lat.), B: external rotators (piriformis, tric. cox., obt. int., quad. fem.), A/B: percentage of PCSA.

	A [cm^2^]	B [cm^2^]	A/B [%]
Brand	124.6	33.6	26.00
Klein und Horsmann	204.5	80.9	39.50
Lube	99.7	45.7	45.70
Pierrynowski (cited in Brand et al.)	127.2	52.4	41.20

As shown in Table 8, the physiological muscle cross‐sectional areas of the external rotators are considerably large, accounting for 26%–45% of the cross‐sectional area of the muscles extending proximally from the greater trochanter (gluteus maximus, gluteus medius, gluteus minimus, and tensor fasciae latae). Therefore, it is very likely that their more horizontal acting forces in the dorsal third of the trochanteric apophysis lead the growth plate in a more horizontal direction (Figure 12).

While in the current work we focused on the spatial orientation of the growth plate interfaces, the thickness of the cartilage could also be included in future analyses. This could increase the accuracy of the inferences about the expected force ratios.

#### 4.2.4 Comparison of juvenile and adult hips

Lovejoy et al. (2002) reported stable AY angles during growth in monkeys. The results of this study only partially support this. In ventral and central positions, the apophyseal growth plate is nearly parallel in juvenile and adult patients. While both patient groups have lower AY angle values in the dorsal direction, this tendency was more pronounced in adults. Thus, the orientation of the growth plate in the dorsal third differs depending on the age of the patient. This is due to the principle “form follows function” (Heimkes, 1997). Since these forces are lower in children due to the smaller muscle volume, the flattening is also less pronounced. As muscle mass increases, the applied force increases (Reeves et al., 2004). The body adapts to the new situation (Löhe et al., 1994).

In the frontal plane, the muscle tracts of the hip abductors experience a greater angular change in the ventro‐dorsal direction in adolescent patients than in the control group of adult patients. The reasons for this are mainly due to the steeper muscle trajectory in the ventral layers. In addition to the small gluteal muscles, the external rotators, especially the obturatorius externus, the quadratus femoris, and the triceps coxae, also show differences between adolescents and adults, with all of these muscles having a steeper course in adolescent hips. This can be explained by the fact that the CCD angle is set much greater in children. All of these muscles attach to the greater trochanter. If the greater trochanter is located more medially, as in the coxa valga, the lines connecting the points of origin and attachment (straight line model) are more vertically oriented. As the CCD angle decreases, the muscle attachment shifts more laterally, and the muscle fibers align more horizontally (Yadav et al., 2017). This means that the change in CCD angle during growth also follows the principle of “form follows function,” which can be explained by the increasing muscle forces during growth. Children who have decreased stimulation of their hip muscles, such as those with cerebral palsy, Duchenne gait, or physical inactivity, tend to develop coxa valga later in life (Heimkes, 2016; Sallam et al., 2015). The alignment of the CCD angle is mainly attributed to the hip abductors (Arnold et al., 2001). In axial view, the trajectories of the hip muscles differ between adolescents and adults only in the quadratus femoris muscle. The steeper adolescent trajectory can be explained by the greater anteversion of the femur in children (Moreland, 1980).

Because of the divergence of the anterior and posterior edges of the growth plate of the greater trochanter on conventional radiographs, reminiscent of the open mouth of a crocodile, we propose the name “crocodile sign.” In clinical practice, we observed a clear correlation of the crocodile sign with the rotational position of the leg. However, we also noted a change in angle between different patients on images with standardized lower limb positioning. Thus, there may be a correlation with torsion of the femoral neck in relation to the distal femur, which could provide an opportunity to infer the extent of ante‐ or retrotorsion from plain radiographs of the pelvis, potentially eliminating the need for costly, time‐consuming, and radiation‐intensive studies such as rotational MRIs and rotational CTs.

## AUTHOR CONTRIBUTIONS

Bernhard Heimkes had the idea for the investigation, created the concept and design of the study, supported the acquisition and interpretation of the data, revised the manuscript critically and supervised the whole process. Christian Ziegler supported the acquisition and interpretation of the data, drafted, revised, and formatted the manuscript and the figures. Ferdinand Wagner supported the acquisition and interpretation of the data, drafted, and revised the manuscript. Boris Holzapfel supported the acquisition and interpretation of the data, drafted, and revised the manuscript. Tobias Geith supported the acquisition and processing of the images, helped with the interpretation of the data, and revised the manuscript. Karoline Alleborn participated in the design of the study, acquired the data, analyzed the images, provided input to the interpretation of the data, and revised the manuscript.

### OPEN RESEARCH BADGES

This article has earned an Open Data badge for making publicly available the digitally‐shareable data necessary to reproduce the reported results. The data is available at https://doi.org/10.5282/ubm/data.397.

## Data Availability

The data that support the findings of this study are available from the corresponding author upon reasonable request.
